# Genome Stability Maintenance in Naked Mole-Rat

**Published:** 2017

**Authors:** I. O. Petruseva, A. N. Evdokimov, O. I. Lavrik

**Affiliations:** Institute of Chemical Biology and Fundamental Medicine, Siberian Branch of the Russian Academy of Sciences, Lavrentjeva Ave. 8, Novosibirsk, 630090, Russia; Novosibirsk State University, Ministry of education and science, Pirogova Str. 1, Novosibirsk, 630090 , Russia; Altai State University, Ministry of education and science, Lenina Ave. 61, Barnaul, 656049, Russia

**Keywords:** Heterocephalus glaber, cancer resistance, genome and proteome stability, DNA repair

## Abstract

The naked mole-rat (*Heterocephalus glaber*) is one of the most promising models used to study genome maintenance systems, including the effective repair of damage to DNA. The naked mole-rat is the longest lived rodent species, which is extraordinarily resistant to cancer and has a number of other unique phenotypic traits. For at least 80% of its lifespan, this animal shows no signs of aging or any increased likelihood of death and retains the ability to reproduce. The naked mole-rat draws the heightened attention of researchers who study the molecular basis of lengthy lifespan and cancer resistance. Despite the fact that the naked mole-rat lives under genotoxic stress conditions (oxidative, etc.), the main characteristics of its genome and proteome are a high stability and effective functioning. Replicative senescence in the somatic cells of naked mole-rats is missing, while an additional p53/pRb-dependent mechanism of early contact inhibition has been revealed in its fibroblasts, which controls cell proliferation and its mechanism of *arf-*dependent aging. The unique traits of phenotypic and molecular adaptations found in the naked mole-rat speak to a high stability and effective functioning of the molecular machinery that counteract damage accumulation in its genome. This review analyzes existing results in the study of the molecular basis of longevity and high cancer resistance in naked mole-rats.

## INTRODUCTION

DNA damage caused by environmental stress and normal metabolic processes occur daily at a frequency raging from 1,000 to 1·106 per living cell [1]. As a result, only 0.00017% of the human genome consisting of 3·109 base pairs is damaged, but lesions in essential genes, such as the genes that code for tumor-suppressor proteins, can significantly disturb cellular function. The efficient DNA repair mechanisms that counteract DNA damage accumulation substantially contribute to genome stability maintenance, which is one of the crucial cellular functions. Accumulation of DNA lesions and mutations increases the risk of cancer and is related to aging [2-4]. The defects in DNA repair mechanisms in humans are associated with a number of hereditary diseases [1-4]. Furthermore, the high conservatism of repair pathways allows one to regard the efficiency of DNA repair mechanisms as one of the underlying reasons behind longevity [2-7]. Only a few experimental studies have focused on the search for a correlation between the activity of DNA repair systems and maximum lifespan [8, 9]. The complexity of these studies and their controversial findings may stem from both the imperfect methods used for activity assessment and improper selection of model systems [10].

The naked mole-rat (NMR, *Heterocephalus glaber*) is one of the most promising models used to study genome maintenance systems, including effective repair of DNA damage. The NMR is the longest living of small burrowing mammals. It’s native to Southeastern Africa (Ethiopia, Kenia, Somalia) and slightly larger than a mouse. NMR colonies are housed in about 60 zoos worldwide and a number of laboratories. It is one of the ~50 known burrowing herbivorous rodents, a representative of the exceptionally rare true eusocial mammals [11]. Due to the keen interest in the NMR, the journal Science named this species “Vertebrate of the Year” for 2013. The lifespan of the NMR can reach 32 years, ten times longer than that of the mouse. For most of its lifespan (at least 80%), this animal shows no signs of aging and retains the ability to reproduce [12-14]. It possesses a very efficient mechanism of resistance to cancer, including cancer induced by different stressors [15]. Initial case reports of cancer in naked mole-rats kept in captivity were published in 2016 [16]. The NMR draws the heightened attention of researchers engaged in the study of the molecular basis of lengthy lifespan and cancer resistance.

Noticeable progress in this area was achieved through research performed using laboratory-generated naked mole-rat lineages and bioinformatics and omics approaches [17-21]. The unique features of the metabolism and its regulation attendant to the NMR have been revealed.

In this review, we have made an attempt to analyze the results of these studies, as well as those of research that employed biochemical and molecular genetic approaches, to paint an overview of the possible features of the DNA repair systems in the NMR.

## STUDYING THE NMR GENOME AND TRANSCRIPTOME USING BIOINFORMATICS APPROACHES

The advances achieved in high-performance whole-genome sequencing have offered us an unprecedented opportunity to reveal the genetic differences of the NMR that underlie the unique traits of this species. An analysis of the data obtained by primary sequencing of the NMR genome revealed a number of typical and important traits; in particular, ones pointing to its enhanced stability [17]. Another version of the genome was subsequently obtained and analyzed [18], and the web portal Naked Mole Rat Genome Resource (http://www.nakedmole-rat.org) was developed. A comparative analysis of the complete NMR and mouse transcriptomes revealed a substantially higher transcription activity for some genes in the NMR. These genes are mainly associated with oxidation/reduction and the mitochondrial function. A record-setting 300- and 140-fold higher expression of the *Epcam *and α*2m *genes coding for the extracellular protein was revealed. The difference between the expression levels of the genes encoding repair proteins in mouse and NMR was not that significant [19].

The first results of a deep sequencing (98.6%) of the genome of a male naked mole-rat were published in 2011 [17]. Back then, the difference in the expression levels of mitochondrial genes and the genes related to the redox system in the NMR and mouse was reported [19]. The sequences of 22,000 NMR genes were predicted using the sequencing data. An analysis of syntenic regions in NMR and human chromosomes identified 750 gained and 320 lost genes; 739 gained and 448 lost genes were revealed in NMR as compared to a mouse. Among the gained genes, 75.5% showed evidence of transcription, while the list of lost genes included many genes related to ribosome and nucleoside biosynthesis functions. Pseudogenes associated with the visual system, olfaction, spermatogenesis, and protein ubiquitination are predominant among all pseudogenes in NMR. Conversion of these genes to pseudogenes (nonfunctional genes) correlates with weakened and suppressed physiological functions in NMR [13] and the accumulation of ubiquitinated proteins with age that is less intense than in mouse [22].

A total of 1.87 million heterozygous single-nucleotide polymorphisms (SNPs) were also identified using the Genome Analysis Toolkit (GATK, https://software. broadinstitute.org/gatk/). The estimated nucleotide diversity (mean per nucleotide heterozygosity) was 7×10^−4^, which is much lower than that in mouse and rat populations and is comparable to the nucleotide diversity in humans. The low level of nucleotide diversity may reflect a low effective size of the NMR population, but it may also be due to a high level of inbreeding, a reduced mutation rate, or high efficiency of the repair systems [17]. Genome stability is believed to correlate with a reduced transposon level. Kim et al. [17] demonstrated that only 25% of the NMR genome is represented by transposon-derived repeats (vs. 40% in the human, 37% in mouse, and 35% in rat genomes).

The *Tep1 *and *Terf1 *genes involved in telomere length regulation belong to the set of positively selected genes in NMR, unlike those in the rat and mouse [23]. Telomere length is short in NMR: the telomeres are shorter than those in laboratory mice or rats and are approximately as long as human telomeres. The *Tert *gene coding for the telomerase catalytic subunit is stably expressed in the somatic cells of NMR at any age. Meanwhile, the telomerase activity is low. A comparative study showed that there is a negative correlation between the levels of telomerase expression and rodent size, since no correlation between telomere length and lifespan has been found 21, 24, 25]. The recent detailed comparison of the genetic structure of telomerase RNA (*hgTerc*) in NMR and other species has revealed two main differences: the A→G replacement in the first loop of pseudoknot P2b-p3 (an equivalent of nucleotide 111 in human telomerase RNA) and the G→A replacement in the CR7-p8b domain (an equivalent of nucleotide 421 in *hTERC*). Two transcription factor binding sites were identified in the promoter regions of the *hgTerc *gene: the ETS family site, which was found to be a conserved element for all the analyzed TER promoters, and the binding site for the SOX17 transcription factor, which was unique to the NMR gene. The absence of one Sp1 binding site was an additional specific feature of the *Terc *gene in NMR [26]. Hence, the NMR *Terc *gene has a unique polymorphism and promoter structure.

The results of a sequencing of RNA isolated from the brain, liver, and kidneys of a newborn, young (4 year-old), and old (20 year-old) NMRs showed that the expression level changes with age in a very small number of genes. In the human brain, the expression level decreased in 33 genes, while increasing in 21 genes [27]. In NMR, the expression level of 32 of these genes did not significantly vary with age: it was stable for 30 genes and increased to some extent in only two genes (*Cyp46a1 *and *Smad3*) [17]. The transcription activity of these human genes decreased with age [27].

Furthermore, Kim et al. [17] performed a bioinformatics analysis of 39 NMR genes encoding a number of proteins associated with G1/S transition, thermogenesis, and the visual function, including cyclin E1 (*Ccne1*), uncoupling protein 1 (*Ucp1*), and γ-crystallin, as well as the proteins that code for the proteins directly involved in DNA metabolism: multifunctional DNA repair enzyme AP endonuclease (APE1), the large subunit of the replication/repair factor RFC1, and topoisomerase TOP2A. TOP2A controls the topologic states of DNA during transcription and, along with TEP1 and TERF1, is part of a 5-protein complex of alternate lengthening of the telomere pathway. A comparison to the orthologs present in the genomes of 36 mammals revealed a divergence in the NMR genome, attesting to the existence of 45 unique amino acid substitutions in the respective proteins [17].

Hence, the first attempted sequencing [17] revealed the important features of the NMR genome, although some of the results were later refined and reconsidered [18, 28, 29]. Thus, the hairless phenotype of NMR was attributed to a replacement of the conserved amino acid residue in the protein associated with hair growth (HR) [17]. This interpretation was based on the fact that such mutations in this codon cause hair loss in mice, rats, and humans. However, two other rodents, the Damaraland mole-rat and guinea pig, also carry this mutation in the *HR *gene but have pelage [29]. The differences between the *HR *genes in NMR and mouse/ humans more likely show the phylogenetic divergence from mouse to humans [29, 30]. The differences in the structure of HAS2 (hyaluronan synthase 2) in NMR are attributed to the exceptional resistance of NMR to cancer [31]. However, some of the presumably important mutations found in the gene encoding HAS2 are identical in several species, including guinea pig. These mutations are not always associated with cancer resistance, and their functional sequelae are unknown as of yet [32]. Interestingly, high-molecular-weight hyaluronans are also synthesized in cancer-resistant long-lived blind mole-rat *Spalax galili*, but its genome carries none of the mutations considered to be key ones in NMR [33, 34]. Furthermore, the conclusion [17] regarding the reduced level of instability source (transportosons) in the NMR genome as compared to those in the mouse and human genomes remains to be adjudicated [28].

A comparative analysis of a group of the genes involved in genome stability maintenance in humans, mouse, and mole-rat has demonstrated that an elevated gene copy number is not typical of the NMR genome [20]. Meanwhile, the *Cebpg *gene coding for the transcription factor involved in DNA repair regulation is represented by three copies; and the *Tinf2 *gene of the shelterin complex component, by two copies. Furthermore, the NMR and human genomes, as opposed to the mouse genome, were found to carry the *Rpa4 *gene coding for an analogue of the second subunit of the RPA protein that consists of three subunits (RPA1, RPA2, and RPA3) and is involved in many processes related to DNA conversions. Full-length coding sequences of this gene were previously revealed only in the genomes of apes and horse [35]. The RPA4 and RPA2 proteins can be expressed simultaneously, while the ratio between their levels depends on the tissue type. The αRPA heterotrimer (an alternative RPA containing the RPA4 subunit instead of RPA2) cannot maintain SV40 replication (the common model to study replication *in vitro*) but exhibits an increased affinity for damaged DNA and participates in the repair and activation of cell-cycle control (the G2/M stages) [36-38].

The higher quality of genome annotation has made it possible to identify ~1,800 non-coding and ~42,000 coding DNA regions and approximately the same amount of proteins using sequencing data. As a result, NMR was found to exhibit a number of features of the gene sequences associated with cancer resistance and aging [18]. Unique replacements in the fragment of the *p53 *gene that encodes the region involved in apoptosis regulation, as well as in the hyaluronan receptor genes CD44 and HMMR, were revealed. Furthermore, NMR p53 carries the PXXP motifs (P – proline and X – any other amino acid), similar to the PXXP motifs in human p53.

Investigation of the genomes and transcriptomes of nine African naked mole rat species has demonstrated that the genes related to tumor suppression, telomere regulation, cell division, RNA repair, and response to stress have been under positive selection in these species [30].

Modern bioinformatics approaches allow one to perform a full-scale targeted comparison of the transcriptomes of gene groups in different animal species. The liver is an organ characterized by a high level of oxidative metabolism and a significant number of spontaneous lesions. MacRae et al. [39] performed a targeted comparison of the expression levels of the genes encoding repair proteins in the liver tissues of long-lived species (humans and NMR) and short-lived mouse. A comparison of a sample consisting of 130 genes revealed that the transcription activity of these genes was higher in the long-lived species. The gene of tumor suppressor p53, the key regulator of excision repair pathways was among the 12 genes whose expression level was at least twice as high both in human and in NMR. Higher expression levels were also shown for the genes encoding the mismatch repair proteins (MSH3) and base excision repair proteins – DNA glycosylase (MUTYH, MBD4, NEIL1, NEIL2 and TDG), the proteins partaking in nonhomologous recombination (NHEJ1, Ku70, DNA polymerase λ – POLL and κ – POLK), and ubiquitin ligase UBE2N.

Most genes encoding DNA repair proteins are constitutively expressed and regulated by post-transcriptional modifications. Nevertheless, transcription of some genes in this group is induced upon genotoxic stress, including the genes coding for the key components of the nucleotide excision repair (NER) pathway: DDB1, DDB2, ERCC1, XPC, ERCC4 (XPF), and ERCC5 (XPG) [40]. A specialized algorithm for signaling pathways [41] was used to demonstrate that the strongest response to genotoxic stress is provided by the pathways controlled by ATM, BRCA1, p53, and PTEN [39].

## EARLY CONTACT INHIBITION

A vast body of results of studies focused on the biochemical features of NMR and aimed at searching for the mechanisms underlying the unique phenotypic traits of NMR, including its cancer resistance, has been published. The unique system of early contact inhibition of cell growth discovered in 2009 is one of these mechanisms [31]. Contact inhibition is a key mechanism that arrests cell division when cells reach a density at which they begin to enter into contact with each other or the extracellular matrix [42]. In humans and mice, regular contact inhibition is mediated by membrane proteins and takes place at an upregulated expression of the cyclin-dependent kinase (CDK) inhibitor p27^Kip1^. P27^Kip1^ binds to cyclin–CDK complexes and arrests cell division at the G1 phase of the cell cycle. The key tumor-suppressor pathways, the Rb and p53 pathways, are activated by products of the *Ink4a *and *Arf *genes [43-46]. Protein p16I^NK4a^, the *Ink4a *gene product, binds to and inhibits CDK 4/6, thus activating Rb [43]. The *Arf *gene product activates p53 by binding to and activating the MDM2 protein. Hence, the *Ink4a* and *Arf *genes play a crucial role in senescence and resistance to cancer [44-48].

**Fig. 1:**
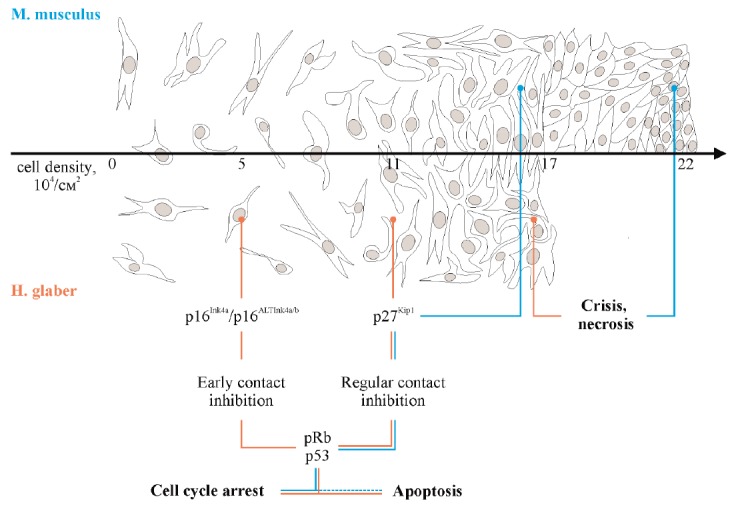
Two tiers of contact inhibition in naked mole-rat *H. glaber *(based on the data presented in papers [31, 49]). In contrast, mouse only has regular contact inhibition.

Replicative senescence is not typical of NMR fibroblasts, but in cell culture, the latter grow slowly and arrest at a much lower density, thus showing hypersensitivity to the emergence of intercellular contacts. It was shown that there is an additional mechanism that controls cell proliferation termed “early contact inhibition” (ECI). ECI in NMR was initially believed to be associated with increased p16INK4a protein levels [31]. This hypothesis was based on the fact that p16INK4a is not expressed in the NMR mutant cells SFMut that spontaneously form after long-term culture and lose their capability of early contact inhibition. Recombinant DNA (plasmid) carrying the genes encoding mutant forms of the large T-antigen antibody SV40 that inactivates either p53 (LTK1; pSG5 LTK1), or pRb (LTKΔ434- 444, pSG5 LTΔ434-444), or the wild-type protein gene (wtLT; pSG5 LT) suppressing the activity of both p53 and pRb were used to demonstrate that, as opposed to mouse fibroblasts, the ability of NMR fibroblasts to ECI after transfection with these DNA decreases when the activity of both suppressor proteins is inhibited. The possibility of standard contact inhibition mediated by p27^Kip1^ only backs up ECI mediated by the kinase inhibitor p16INK4a [31]. Later, it was shown using RNA sequencing data that the protein termed pALTINK4a/b appears in cultured NMR cells and tissues upon expression of the product of alternative splicing of the *p15a, p15b *genes and the *Ink/Arf *locus. The pALTINK4a/b protein was revealed in neither mice nor humans. Expression of pALTINK4a/b is induced upon ECI and under stress, such as UV or ionizing radiation, loss of adherence to the substrate, and oncogene expression. Furthermore, pALTINK4a/b is more efficient at inducing cell cycle arrest, thus leaving more time for the cells to overcome the consequences of genotoxic stress, including DNA-damage repair before replication starts. The two-tiered contact inhibition typical of NMR cells (as opposed to mouse and human cells) may contribute to the maintenance of the stability of its genome [49] (*Fig. 1*).

## HIGH-MOLECULAR-WEIGHT HYALURONIC ACID AND ONCOTRANSFORMATION OF NMR CELLS

In accordance with the data reported in [32], early contact inhibition is related to ultra-high-molecular-weight (6–12 MDa) hyaluronans (HA, hyaluronic acid), which are synthesized in NMR tissues and cells and released into the extracellular space. This polysaccharide was previously better known as a component of the extracellular matrix associated with inflammation and cancer. HA fragments of different molecular weights vary in their functions: medium-sized molecules (30–500 kDa) can stimulate cell division, while smaller fragments ( < 50 kDa) can stimulate their migration. Short HA fragments bind to HA receptors, such as CD44 and HMMR, induce inflammation, and activate the signaling pathways that promote survival, migration, and invasion of both tumor and normal cells. Normal human body fluids contain HA 1–8 MDa [50, 51]. In NMR, ultra-high-molecular-weight molecules accumulate due to the low activity of its hyaluronidases and high processivity of hyaluronan synthase 2 (HAS2), whose active site has a specific structure. Substitution of asparagine residues at positions 188 and 301 with serine in HAS2 facilitates the synthesis of ultra-high-molecular-weight HA polymers. Disruptions within the signaling pathways relieving the limitations for initiation of mouse fibroblast oncotransformation do not cause a transformation of NMR cells. If synthesis of high-molecular-weight HA is arrested as a result of HAS2 knockdown or HA degrades rapidly due to an elevated expression of hyaluronidase, NMR cells become susceptible to transformation [32].

## EARLY CONTACT INHIBITION AND THE NEW TYPE OF SENESCENCE IN NMR CELLS: Arf SUPPRESSION-INDUCED SENESCENCE

Discovered in 2009, the phenomenon of early contact inhibition of fibroblast growth in NMR remains interesting to researchers. In addition to the recently revealed pALTINK4a/b protein, the product of expression of the alternatively spliced form of *Ink4 *partaking in ECI [49], another new effect specific to NMR cells has been discovered: *Arf *suppression-induced senescence. The coding sequences of the NMR *Ink4a *and *Arf H* genes were identified using conventional cloning procedures and subsequent Sanger sequencing; lentiviral vectors carrying these genes and high-specificity polyclonal antibodies against the respective proteins were constructed. Endogenous *Ink4a *and *Arf *expression in NMR fibroblasts was shown to be upregulated following exposure to DNA-damaging factors or serial passaging [52]. The upregulated *Ink4a *or *Arf *expression caused cell cycle arrest in NMR fibroblasts. Hence, it was experimentally proved that the genes involved in producing the effect of early contact inhibition play a conserved function of cell cycle inhibitors in NMR [52]. These results were used when studying the mechanisms that suppress tumor development from induced pluripotent stem cells (iPSCs) in NMR [53]. Tumorigenicity of iPSCs was tested for its teratoma-forming potential. NMR iPSCs transplanted into a tested mouse, unlike a number of other stem cells, did not form teratomas; i.e., they were not tumorigenic. This unique feature is based on species-specific activation of the *Arf *oncosuppressor gene and a unique frameshift mutation in the *RAS *(*ERAS*) oncogene expressed by stem cells. The upregulated expression of the *Arf *gene in mouse iPSCs noticeably reduced their tumorigenic potential. The mechanism related to NMR cells that can protect iPSCs and somatic cells against *arf *suppression and tumor formation was found. A special type of senescence, *Arf *suppression-induced senescence, was also revealed in NMR iPSCs. The *Arf*-dependent senescence specific to NMR can act as a backup protection method inducing cell senescence and following death by suppressing *Arf *expression in the cells where this gene used to be suppressed under stress [53].

## APOPTOSIS

Apoptosis is one of the mechanisms used for resisting the oncotransformation of cells. The ability of NMR cells to undergo apoptosis in response to genotoxic stress has been insufficiently studied. When investigating the mechanism of ECI, Seluanov et al. [31] demonstrated that the spontaneous apoptosis level in NMR fibroblasts is low (no higher than 7% in skin fibroblasts and 15%, in cultured lung fibroblasts) and is characterized by specific regulation. The count of apoptotic cells in these cultures abruptly increased approximately twofold after transfection with plasmids carrying the genes coding for the mutant forms of the SV40 large T-antigen, pSG5 LTK1 and pSG5 LTΔ434-444. Transfection of NMR fibroblast cultures with plasmid pSG5 LT carrying the wild-type gene reduced the count of apoptotic cells in them below the control level, while LT had no effect on mouse fibroblasts [31]. In a mouse and humans, apoptosis is also induced to a certain extent when the cell cycle regulator pRb loses its activity [54, 55]. In order to elucidate the mechanism ensuring inhibition of fibroblast growth in NMR upon inactive p53, NMR fibroblasts transfected with these recombinant plasmids were cultured in the presence of the caspase inhibitor Z-Vad-FMK. Growth of fibroblasts transfected with pSG5 LTΔ434-444 increased in the presence of the apoptosis inhibitor. The mutant protein LTΔ434-444 inactivates pRb, thus disturbing the mechanism of cell cycle arrest. A combination of pRb inactivation and apoptosis inhibition in the presence of Z-Vad-FMK results in cell growth, to achieve high confluent density. The growth pattern of cells transfected with pSG5 LTK1 in the presence of the apoptosis inhibitor remained unchanged. Z-Vad-FMK and LTK1 inactivate p53, while pRb remains active: it induces cell cycle arrest and controls cell proliferation [31].

The necrotic cell death pathway is also typical of the cancer-resistant *Spalax *genus of blind mole rats (*Spalax ehrenbergi *and *S. galili*) [56]. In *Spalax*, p53 differs from that in most related mammals by having an arginine-to-lysine substitution at position 174. This specific mutation is frequently detected in human tumors [57]. The arginine-to-lysine substitution affects the properties of the DNA-binding domain of p53. The protein carrying this substitution can induce cell cycle arrest but cannot induce apoptosis. R174K mutation in p53 reduces its ability to activate the apoptotic cascade and activates immuno-inflammatory processes stimulating the development of necrosis induced by interferon-β1 [55, 56]. Nevertheless, the pathway associated with the activity of p53 is also needed for necrotic cell death in *Spalax* [57-60]. As opposed to *Spalax*, the arginine residue occupies position 174 in *H. glaber *p53, as well as in normal human and mouse cells [18].

The study by Salmon *et al*. focused on the effect of toxic stressors on NMR fibroblasts demonstrated that these cells are more resistant to methyl methanesulfonate, paraquat, and low-glucose media but more sensitive to H_2_O_2_, UV light, and rotenone compared to mouse fibroblasts [61]. Labinskyy et al. compared the apoptotic response of the cultured arterial endothelial cells of NMR and laboratory mouse to oxidation with H_2_O_2_ at a concentration ranging from 10-6 to 10-3 M and heat (42°C). The apoptotic response of NMR cells to exposure to H_2_O_2_ was 3- to 10-fold weaker, while their resistance to heat was higher than that in mouse endothelial cells [62].

## TRANSLATIONAL FIDELITY AND SPLIT 28S rRNA

Translational fidelity is one of the key features of the functioning of key NMR systems. With the translation rates close, the number of misincorporated amino acids in NMR fibroblasts is fourfold lower than that in mouse fibroblasts [63]. The translational fidelity in NMR is attributed to the fact that 28S rRNA split into two fragments (that is what NMR 28S rRNA specimens look like after electrophoresis under denaturing conditions) optimizes the folding and/or dynamics of the large ribosomal subunit [63]. Comparison of transcriptomes in a number of rodents showed that degradation of NMR 28S rRNA results from the deletion of a fragment of specific sequence located in the D6 domain of 28S pre-rRNA [64]. In NMR and the Talas tuco-tuco (*Ctenomys talarum*), these sequences are characterized by a high degree of sequence conservation. Its 28S rRNA also looks like it is split, but highly accurate protein biosynthesis is not typical of the tuco-tuco [64]. Quite a few species with splitting in RNA are known, but no correlation with their lifespan was revealed. It is also unclear if 28S rRNA is split as a result of specific splicing and fragments are linked into one structure by hydrogen bonds only or if splitting is an artifact emerging under high temperature during RNA isolation or analysis [65-69]. Therefore, attributing the exceptional translational fidelity in NMR to the structural features 28S rRNA is controversial. The high accuracy of protein biosynthesis undoubtedly contributes to the stability of the NMR proteome; however, the features of the underlying molecular mechanisms are yet to be studied. In particular, the first translation stage that is significantly responsible for the accuracy of protein synthesis (tRNA aminoacylation) remains completely unstudied in NMR [70].

## OXIDATIVE DAMAGE AND PROTEIN STRUCTURE STABILITY

The oxidative stress theory considers the accumulation of oxidative damage in cells to be one of the factors behind aging. For this reason, the questions regarding the level of oxidative damage and the features of the mechanisms of antioxidant protection in the “long-live” NMR draw researchers’ attention.

Proteins are the main target where oxidative damage emerges. Oxidative events may disrupt the protein structure and functions, in particular by inactivating enzymes and facilitating the formation of protein aggregates containing covalent cross-links. The cysteine thiol groups are characterized by high sensitivity to oxidation, since they can form both reversible (disulfide S–S, sulfenic acid) and irreversible lesions (sulfinic and sulfonic acids) [22]. Other common types of oxidative damage to proteins include carbonylation, irreversible modification of the side chains of proline, arginine, lysine, threonine, cysteine, and histidine residues [71]. Lysates of tissues of different organs of NMR and laboratory mice of respective physiological age are mostly used as model systems to study oxidative damage in proteins [22, 72-76]. The level of oxidative damage in cysteine, the carbonylated protein level, the effect of oxidative damage on the protein structure and function and the activity of a number of the enzymes involved in resisting accumulation of oxidative damage were studied.

Comparison of the activities of glutathione synthetase, catalase, superoxide dismutases, and glutathione peroxidase (GPX1) demonstrated that the activity of all enzymes but GPX1 in liver extract from a young naked mole-rat was 1.3- to 2-fold higher than that in the extract from the liver of a C57BL/6 mouse of respective physiological age. GPX1 activity in the NMR extract was lower by almost an order of magnitude [72]. In accordance with more recent data, the levels of mRNA *Gpx1 *and the respective protein also abruptly decrease in NMR [19, 73].

According to [22], the level of free thiol groups and reversible oxidative damage such as S–S and sulfenic cysteine derivatives in the proteins of young NMR is 1.6-fold higher than that in mouse proteins (C57BL/6). Furthermore, the level of oxidative damage to cysteine in mice increases 3.4-fold with age and the levels of irreversible oxidative damage in cysteine and carbonyl lesions increase, while such changes were not observed in NMR [22, 72-76]. This demonstrates that the performance of the systems counteracting oxidative stress in NMR is more efficient.

An analysis of the levels of protein carbonylation in NMR and mouse tissues demonstrated that triose phosphate isomerase (TPI) and peroxy redoxin 1 (Prdx1) are the main targets for carbonylation in all samples. NMR proteins are characterized by a 1.5-old higher level of carbonyl damage but better retain enzymatic activity.

The level of carbonyl damage in NMR proteins is 1.5-fold higher, but the proteins better retain their enzyme activity. The specific activity of TPI in the cytosol fraction of a NMR kidney tissue lysate was three times higher than that of mouse. Furthermore, NMR TPI and Prdx1 form fewer covalently cross-linked protein oligomers under oxidative stress (ascorbate/Fe^2+^) [73, 74]. 4,4’-Dianilino-1,1’-binaphthyl-5,5’-disulfonic acid (BisANS) was used as a nonpolar fluorescent probe that interacts with hydrophobic amino acid residues on the surface of protein globules to demonstrate that NMR proteins are much more resistant to the denaturing effect of 1 M urea than mouse proteins. In particular, glyceraldehyde 3-phosphate dehydrogenase (GAPDH), whose active site contains thiol groups, retains 60% of its activity in NMR, as opposed to 10% in mouse GAPDH [22].

Comparison of the distribution of carbonylated proteins over subcellular fractions in long-lived (including NMR) and short-lived mammals demonstrated that the relative level of proteins with oxidative damage in the nucleus in long-lived animals is lower than that in the cytoplasm [9, 76]. This gave grounds for suggesting an reverse correlation between the level of oxidative damage to nuclear proteins and lifespan [76]. However, no detailed studies have been performed to verify this speculation. No data on the level of damage to the proteins involved in DNA repair are available.

The actual situation may be disguised by the results of evaluations that do not distinguish between the types of damage or damaged molecules (proteins or DNA) originating from different cellular compartments. Lack of consistency in the nature of the methods and agents used in different publications also impedes any analysis. One of the reasons for this can be the fact that a high level of oxidative damage is typical of only certain molecules (molecular classes) and/or cellular compartments [76].

Furthermore, the data regarding the antioxidant status are controversial. Thus, a lack of consistency was observed for the GSH levels in NMR tissue evaluated in different studies: it was reported to be 1.4-fold lower than that in mouse [77], while the level reported in [22] was 1.4-fold higher. This discrepancy does not allow one to compare the antioxidant status of these organisms. Furthermore, the phenomenon of eusociality may also affect the results of the experiments with organ tissue extracts and body fluids from NMR [78].

## THE UBIQUITIN-PROTEASOME SYSTEM AND THE UNIVERSAL PROTEASE INHIBITOR, ALPHA-2-MACROGLOBULIN

The ubiquitin-proteasome system plays a crucial role in maintaining the required level of active proteins with a proper structure (proteostasis) in the cell [79].

Evaluation of the proteolytic activity in combination with the results of Western blotting revealed a higher chimotrypsin-like (ChT-L) and trypsin-like (TL) protease activity in 26S and 20S proteasomes in liver tissue extracts of NMR*. *Specific ChT-L activity of NMR proteasomes was shown to be 3–5 times higher than that of mouse proteasomes [80]. Most of this activity is provided by the activity of 26S proteasomes. Furthermore, 20S proteasomes can perform ubiquitin-independent hydrolysis of proteins containing oxidative damage, such as carbonylated proteins [79]. This may contribute to the maintenance of stable functioning of the NMR proteome in which the level of protein ubiquitination is low and does not increase with age. The levels of the 19S regulatory subunits and immunoproteasome catalytic subunits (β5i and β2i) in NMR are also higher than those in mice [80]. Furthermore, the expression level of the key chaperons HSP72, HSP40, and HSP25 is higher in NMR. Two of these chaperons are components of the so-called cytosolic protein factor that protects proteasomes against inhibitors and increases their efficiency [81]. The increased peptidase activity and involvement of chaperones in the protection of proteasomes against inhibitors observed in NMR are chaperone functions that had been previously unknown. All these facts could be indicative of the fact that NMR is characterized by high proteome quality control.

The multifunctional blood plasma protein alpha- 2-macroglobulin (α2m) is also associated with proteostasis maintenance. Human α2m can bind to various cytokines, growth factors (TGF-β1, TNF-α, and IL-1β), and it is a universal inhibitor of proteinases (trypsin, chymotrypsin, elastase, and metalloproteinases). Binding of α2m-proteinase complexes to the LRP1 (CD91) receptor triggers their quick elimination from the blood and tissues via receptor-mediated endocytosis. This protein is believed to act as a chaperone preventing protein aggregation and to facilitate the retention of zinc in cells (in humans, reduction of the zinc level with age is accompanied by the development of a number of diseases) [82-85]. The level of transcription of the gene coding for α2m in NMR liver is elevated 140- fold compared to that in mouse liver [19]. Blood plasma concentration of the α2m protein in NMR is 2–3 times higher than that in humans. This fact is potentially responsible for the proteolytic activity of blood plasma in NMR, which is lower compared to that in humans [86].

Another important feature of NMR is the constant activity of the signaling pathway regulated by the Nrf2 factor, which activates the transcription of over 200 genes involved in the antioxidant and anti-inflammatory response of the organism to endogenous and exogenous stressors [87].

## CONCLUSIONS: STABLE GENOME, A STABLE GENE EXPRESSION LEVEL, STABLE PROTEOME, AND EFFICIENT DNA REPAIR

Efficient “functioning” of DNA repair systems is believed to be one of the basics of genome stability maintenance. The typical features of the NMR genome include an increased stability of its structure and function, which are maintained during the entire lifespan. Its protein system (the proteome) is also stable. Translational fidelity, upregulated expression of key chaperons, and permanently active proteasomes in combination with a high α2m expression level facilitate the maintenance of a pool of efficiently functioning proteins in NMR cells. Resistance to denaturing conditions and the ability to retain their functional activity under permanent oxidative stress were experimentally proved for a number of NMR proteins. All the aforementioned factors and the upregulated expression of a number of genes coding for repair proteins, as well as the intensity of the response of the signaling pathways to damage, provide grounds to expect a high efficiency of the DNA repair system in NMR. In particular, this speculation is consistent with the results of studies using cells of mammals with different lifespans. The rate of UV-induced DNA synthesis in the fibroblasts of the white-footed mouse *Peromyscus leucopus *is 2.5-fold higher than that in mouse (*Mus musculus*) fibroblasts [8]. UV-induced damage in the fibroblasts of long-lived Snell dwarf mice is repaired more efficiently than in the fibroblasts of a mouse with a normal lifespan [9]. Comparison of the activities of poly(ADP-ribose) polymerases (PARPs) in the mononuclear blood leukocytes of 13 mammalian species revealed a positive correlation between the PARP activity level and the maximum lifespan typical of these mammals. In particular, PARP activity in human cells was shown to be five times as high as that in rat cells. Meanwhile, no difference in the levels of the respective proteins was observed and no significant poly(ADP-ribose) polymer degradation was detectable under the experimental conditions, ruling out any interference by poly(ADP-ribose) glycohydrolase (PARG) activity. A hypothesis that a higher poly(ADP-ribosyl)ation capacity might contribute to the efficient maintenance of genome integrity and stability in long-lived species was put forward [88].

It is quite possible that the activity of the poly(ADP-rybosyl)ation processes that regulate different repair mechanisms [89] is also elevated in the extremely long-lived NMR; however, no experimental evidence to this fact has been obtained thus far.

The unique phenotypic traits of NMR [90] are obviously based on the structural and regulation features of its genome and proteome.

**Fig. 2:**
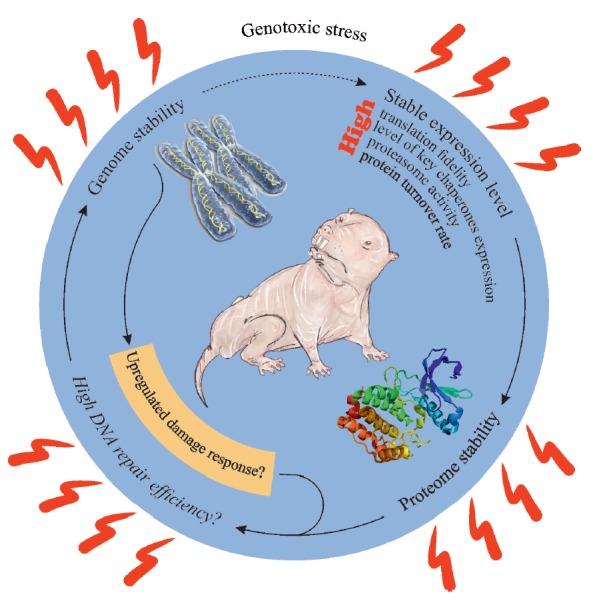
Stable expression level, proteome stability and upregulated damage response maintain *H. glaber *genome stability upon genotoxic stress.

Model systems of different complexities are employed to study these features using an increasingly broad range of methods [91]. Ma et al. [92] have recently conducted a study using fibroblasts from 16 mammalian species to demonstrate that upregulated expression of the genes encoding the proteins associated with DNA repair is typical of long-lived mammals. Modeling and analysis of the stability of the genetic networks linking age, stress resistance, and decelerated physiological senescence have demonstrated that the stability of the simplest model genetic network increases sharply when such a parameter as “efficient repair” is added to the calculations. Furthermore, according to modeling results, the contributions of DNA repair and the processes ensuring the presence of efficiently functioning proteins in the cell (proteostasis maintenance and proteome repair) to the stability of the genetic network are equally significant, while these processes are interrelated [7].

Hence, there is good reason to believe that the molecular machinery counteracting the accumulation of damage in the NMR genome, including the mechanisms of DNA repair, is very efficient. We have made an attempt to illustrate this conclusion with a scheme shown in *Fig. 2*. However, the lack of studies focused on apoptosis induction under various genotoxic stressors and experimental data regarding the function of DNA repair systems leave a gap in knowledge regarding the real contribution of these processes to the longevity and cancer resistance of NMR. In this connection, a comparative evaluation of the functional activities of DNA repair systems is a rather important and topical task.

## Abbreviations

H. glaber: Heterocephalus glaber
Spalax: Spalax galili
M. musculus: Mus Musculus
MLS: maximum life span
OD: oxidative damages
GPX1: glutathione peroxidase
GSH: reduced glutathione
ECI: early contact inhibition
HA: hyaluronic acid
HAS2: hyaluronan synthase 2
PARP: poly(ADP-ribose) polymerase
PARG: poly(ADP-ribose) glycohydrolase
NMR: naked mole-rat
